# 

**Published:** 1886-12

**Authors:** 


					LIST OF SUBSCRIBERS
TO
(The Bristol flDefcico^Gbtntrgtcal 3ournal.
jBnolanfc.
CHESHIRE.
EGREMONT. MACCLESFIELD.
T. W. A. Napier T. S. Sheldon
CORNWALL.
BODMIN. LOSTWITHIEL. REDRUTH.
T. Mudge C Row T B Carlyon
W- R?w J. S. Hichens
calstock. J- H. Sewart g. A. Michell
H. T. Woodd NEW QUAY.
T. Boyle ST- austell.
camborne. \y_ Urwick W. Mason
J" Pau" PADSTOW.
ST. JUST.
camelford. H- F" Mar'ey R B Sear]e
E. Pearce penzance.
W. S. Bennett saltash.
polruan. Runnalls
A. A. Davis w H Torbock
TRURO.
LISKEARD. PORT ISAAC.
J. B. Jackson
J. H. Jenkins C. Williams. E. Rundle
DEVONSHIRE.
ASHTON. CHAGFORD. EXBRIDGE.
S. Thomson A. D. Hunt G. F. Sydenham
BAMPTON. CHILLINGTON. EXETER.
T. A. Guinness F. H. Clarke J. M. Ackland
BARNSTAPLE. C HUD LEIGH. A/wI'lSmpe
t H- Gamble J.A.Watson L. Shapter
,,,J'. 2.rP?r ? E. B. Steele-Perkins
W. A. G. Laing combe martin. L. H. Tosswill
BEER ALSTON. G- H- Manning honiton.
A" R- Stecl DAWLISH. X. w. Shortridge
bideford. A. de VV. Baker
F. M. Cann horrabridge.
VV. H. Ackland
J. Thompson
A. P. Prowse
DEVONPORT.
BLACK TORRINGTON. G. T. Rolston KINGSBRIDGE.
H. H. Parsloe J- R- Rolston W. H. Webb
BRIXHAM. DOLTON. LYNTON.
G. C. Searle S. Michell F- C. Berry
BROADCLYST. DUNSFORD. MORCHARD BISHOP.
J. Somer R. Riddell W. J. C. Nourse
290
SUBSCRIBERS.
DEVONSHIRE (Continued).
POOLE. THORNCOMBE.
J. McNicoll T. A. Palm
PORTLAND. WEYMOUTH.
G. H. Lilley R. W. Carter
NEWTON ABBOT. ST. MARY CHURCH. TAVISTOCK.
3: ^a^on T. Finch M. T. Leamon
W. G. Scott
OTTERY ST. MARY. SALCOMBE. TEIGNMOUTH.
F. A. Gray A. H. Twining R- Gibbs
PAIGNTON TIVERTON.
T Awln?r SIDMOUTH. J. S. Smith
J. Alexander B G PulHn
PLYMOUTH. T. H. S. Pullin TORQUAY.
R. H. Clay W- Powe11
F. W. P. JagO SOUTH MOLTON. WINKLEIGH.
Medical Society E. Furse J. H. Norman
DORSETSHIRE. /
BEAMINSTER. PARKSTONE. STURMINSTER NEWTON.
T.P.Daniel J. R. Philpots J. C. Leach
BRIDPORT.
G. M. Evans
CEKNE ABBAS.
E. W. Kerr
DORCHESTER.
F. B. Fisher Sherborne. wimborne.
P. W. MacDonald VV. H. Williams A. J. H. Crespi
ESSEX.
SOUTHEND.
E. E. Phillips
GLOUCESTERSHIRE.
AVONMOUTH.
J. C. Heaven
BRISTOL.
G. Adams N. C. Dobson G. A. Imlay
J. B. Adams C. H. Dowson G. W. Isaac
O. Andrews W. Dowson W. P. Iieall
G. F. Atchley _ E. L. W. Dunbar F. P. Lansdown
T. G. L. Baretti T. F. Edgeworth A. E. A. Lawrence
B. J. Baron C. Elliott H. Lawrence
J. Beddoe J. F. Elwin H. Laxton
A. E. Blacker J. F. Evans E. L. Lees
A. F. Blagg J. Ewens \V. E. Lloyd
E. C. Board W. J. Fawcett F. T. B. Logan
J. Broom R. G. Fendick W. C. Luffman
A. Broster E. L. Fox W. C. Lysagln
G. F. Burder w. J. Fyffe H. Marshall
J* 1P-Bush G. Gardiner C. E. Matthews
A- Carr General Hospital T. O. Mayor
_ iv-F- garter A. N. G. Gibbs J. S. Metford
J. P. Challacombe G. A. Gloag J. Milne
J.M.Clarke W. C. Goodden Museum and Library
E. W. Coathupe W. G. Grace C. Newman
R- W. Coe L. M. Griffiths W. H. C. Newnham
T. \ . Coker C. D. G. Hailes J. Newstead
T J- Colmsin A. J. Harrison T. D. Nicholson
G. G. Corbould W. H. Harsant J. A. Norton
F. R. Cross S. Harwood T. Ci Norton
A. G. Cunningham C. F. Hawkins J. H. Parry
J* Dacre A. G. Hayman T. C. Parson
J. G. Davey C. K. C. Herapath J. D. F. Parsons
D. S. Davies H. E. Hetling G. C. P. Pauli
H. R. Dew F. F. Hills \V. J. Penny
SUBSCRIBERS. 29I
GLOUCESTERSHIRE (Continued).
Bristol (continued).
J. H. Perkins J. E. Shaw J. G. Swayne
C. F. Pickering E. J. Sheppard S. H. Swayne
\V. E. Pountney W. E. Shorland J. Taylor
A. Prichard E. M. Skerritt G. S. Thomson
A. W. Prichard G. M. Smith H. C. Thurston
J. E. Prichard J. G. Smith W. J. Tivy
A. Pring R. S. Smith H. Waldo
A. B. Prowse S. Smith C. H. Wallace
G. Rogers W. H. Spencer E. H. Warner
W. B. Roue G. M. Stansfeld J. H. Wathen
Royal Infirmary C. Steele T. Webster
C. K. Rudge G. Stock G. G. D. Willett
H. T. Rudge F. W. Stoddart P. W. Williams
J. Swain
CHALFORD. FKAMPTON-ON-SEVERN. STAPLETON.
F. C. Palmer T. Watts H. A. Benham
CHELTENHAM. GLOUCESTER.
L
CHIPPING SODBURY
G. Thompson
STOKE BISHOP.
J. W. Bramwell R- W. Batten
J.Bubb F.T.Bond J.Stewart
Cheltenham Library E. D. Bovver JJ. Walsh
J.E. Gabb E. I. Rowe F. I*. Welsh
J. C. Gooding A. M. Sydney-Turnei stonehouse.
C. J. Newton H. H. Tomkins w?tt#?re
Winterbotham J- P. Wilton G. T. BAVatters
STOW-ON-THE-WOLD..
HAMBROOK. TWncr
A. Grace ?E.Crossman E' 8
W. F. B. Eadon stroud.
CINDERFORD. HAWKESBURY-UPTON. A.S.Cooke
W. C. Heane ... t ~ ? E. A. Howsin
W. I. Cox W. H. Paine
cirencester. kingswood. F. W. Storry
E. C. Cripps tt orace tetbury.
C-RH00ker LYDBROOK.
COLEFORD. H g pletcher THORNBURY.
L. B. Trotter E. M. Grace
lydney. W. G. Salmon
downend, a. S. Currie T. H. Taylor
H. Skelton marshfield. westbury-on-trym.
dursley. J- Reader H. Ormerod
F. J. Joynes moreton-in-marsh. winchcombe.
L. K. Yelf C. Penruddocke
fairford.
J. Cornwall shurdington. winterbourne.
J. Hitchman J. Lambe R. Eager
fishponds. staple hill. wotton-under-edge
H. R. Mead W. Murray D. H. Forty
HAMPSHIRE.
BOURNEMOUTH. LYMINGTON.
D. C. Embleton J- Rendall
V. Fasulo southsea.
E. P. Philpots H Rund)e
HEREFORDSHIRE.
HEREFORD. ROSS.
W. C. Whitfeld J- W. Norman
2g2 SUBSCRIBERS.
HERTFORDSHIRE.
ST. ALBANS.
A.H. Boys
LANCASHIRE.
LIVERPOOL. MANCHESTER. POULTON-LE-FYLDE.
Medical Institution. J. Scott P. H. Day
MIDDLESEX.
ENFIELD.
VV. B. Kesteven
LONDON.
L. S. Beale J. M. Fothergill Royal Medical and
E. Carnall G. S. Gregory Chirurgical Society
H. Cooper C. M. Jessop \y. j. Seward
TOTTENHAM.
W. H. Plaister
MONMOUTHSHIRE.
ABERGAVENNY. MONMOUTH. PONTYPOOL.
S. H. Steel G. Wilson S. B. Mason
W. D. Steel rhymney.
NEWPORT. T. H. Redwood
CAERLEON.
W. Basset risca.
G. B. Robathan
J.A.Morris H.R.Hudson
O. E. B. Marsh
EBBW VALE. A G. Thomas TREDEGAR.
J. W. Davies H. E. Williams G. A. Brown
NORTHAMPTONSHIRE.
KETTERING.
R. E. Burges
OXFORDSHIRE.
OXFORD.
R. W. Doyne
SOMERSETSHIRE.
BANWELL.
A. J. Bisdee
BATH.
W. B. Beatson H. F. A. Goodridge T. P. Lowe
F. H. Blaxall T. B. Goss F. Mason
E. J. Cave F. K. Green G. Norman
T. Cole F. P. Hoblyn M. W. H. Russell
F. S. Cowan H. C. Hopkins R. J. H. Scott
A. E. W. Fox L. J. King E. Skeate
H. W. Freeman J. K. Spender
B EC KINGTON. BRUTON. CLEVEDON.
W. G.Evans P. Stockwell T. Davis
BURNHAM. G. F. P. Pizey
bridgwater. j} ^ Fraser Skinner
F. J. C. Parsons A. W. C. Peskett
J. B. Sincock Cheddar.
R. W. Statham
BRISLINGTON.
B B Fox CHEW MAGNA. DULVERTON.
C. H. Fox E. T. Hale R. J. Collyns
COMPTON MARTIN.
W. F. Lovell
SUBSCRIBERS. 293
SOMERSETSHIRE (Continued).
FROME. NORTH PETHERTON. WELLINGTON.
. F. H. Burroughs W. J. Todd J. Meredith
F. Parsons T. E. P. Prideaux
F. E. Pearse portishead.
Glastonbury. Weatherly wells.
J. A. Bright ridgway. Fairbanks
ilminster. W. Duncan
bradford-on-avon.
WILTSHIRE.
3aianb.
ANTRIM.
BELFAST.
J. W. Browne
A. L. Wade
C" MUnd6n SHEPTON MALLET. WESTON-SUPER-MARE.
keynsham. g Hyatt G. E. Alford
N. Crisp G. B. Fraser
J. Lodge south petherton. E. F. Martin
R.W.Thomas W.H.Walter W. H.G.Phelps
G. F. Rossiter
langport. street. R- Roxburgh
J. Morgan A Clarke W- S' wicksteed
LONG ASHTON.
T Fuller TAUNTON. WINCANTON.
H.J. Alford J. B. Colthurst
mells. j, Carey
G. Terry w. Liddon wrington.
MIDSOMER NORTON. TEMPLE CLOUD. W. ColllUS
P'acker T. Martin Vitt?
A. Waugh YATTON.
twerton-on-avon. A. de C. Lyons
P. S. H. Colmer
MILVERTON.
C.Randolph J. Wigmore
MINEHEAD. WEDMORE.
T. Clark J. Ford C. J. Marsh
T. J. Ollerhead R. P. Tyley E. Vernon
SURREY.
CHERTSEY.
W. A. Moynan
WARWICKSHIRE.
BIRMINGHAM.
L. Tait
W. D. Lovell C. Clay J. B. Fry
W. Howse
BURBAGE. I.ACOCK. j C Maclean
A. E. N. Browne J. H. Crisp
J r TROWBRIDGE.
calne. malmesbury. G. C. Tayler
W. S. Batten R. Kinneir N- V. Wise
CHIPPENHAM. SALISBURY. WARMINSTER.
G. H. Daly H. Coates J. Hinton
B. C. Gowing R. L. Willcox
DEVIZES- L. S. Luckham
T. B. Anstie H. J. Manning w o
C. Eddowes T. Sanctuary W. Pippette
DOWNTON. STRATTON ST. MARGARET. WOOTTON BASSETT.
G. W. Whiteley J. Fernie J. M. Kirkman
294 SUBSCRIBERS.
Scotlanb.
EDINBURGHSHIRE.
EDINBURGH.
Y. J. Pentland
Wales.
BRECONSHIRE.
BRECON. BRYNMAWR. SENNY BRIDGE.
D. V. Rees G. H. Browne VV. R. Jones
TALGARTH.
W. Howells
CARDIGANSHIRE.
ABERYSTWITH. LAMPETER. NEW QUAY.
R. Gilbertson E. C. Davies E. R. Jones
CARMARTHENSHIRE.
CARMARTHEN. FERRYSIDE. LLANELLY.
G. J. Hoarder P. Williams D. J. Williams
PENCADER.
E. Harries
GLAMORGANSHIRE.
ABERAMAN. CARDIFF. MF.RTHYR TYDVIL.
J. B. James A. P. Fiddian J- L. W. Ward
G. W. Grote penarth.
A,..rA?. MfS'Srt'y A. Devonald
E Jones W. Morgan reynoldstone.
W. Price H. V. Ellis
BRITON FERRY. q ^ Vachcll SWANSEA.
J. Symes H. R. Vachell L. Barnett
G. Padley
I. A. Rawlines
CAERPHILLY. cowbridge. , J. S. H. Roberts
J. Lewellyn J.W.Phillips J.Thomas
MONTGOMERYSHIRE.
LLAN SAIN TFFRAID.
C. Edwardes
INDEX.
Ackland, J. M.? Facial neuralgia and
dental irritation, 28.
Alimentary tract, Cancer of the?
F. B. Jessett (Review), 275.
Alpine winter?A. T. Wise (Review),
142.
Anaemia, Case of pernicious?Dr. T.
Cole, 231.
Aneurism,Intra-thoracic (Extract),45.
Aneurism of aorta, Case of?Dr. R.
Shingleton Smith, 233.
Aspirator in retention of urine, The
use of the?Dr. W. Fairbanks, 242.
Astigmatism, The cause of regular?
(Extract), 204.
Atropine conjunctivitis (Extract), 129.
Bacteriology, Practical ? E. M.
Crookshank (Review), 67.
Baron, Dr. B. J.? Excoriationes na-
rium, 52: the laryngoscope in
medicine, 145.
Bath, Contrexeville and the lime
sulphated waters?Dr. J. Mac-
pherson (Review), 137.
Beddoe, Dr. J.?The Races of Britain
(Review), 64.
Bell, F. J.?Comparative anatomy
and physiology (Review), 69.
Benham, Dr. H.?Calculus on foreign
body in bladder, 38.
Brain, Diseases of, the?Dr. W. R.
Gowers (Rcviei;'), 60.
Brain, Operative surgery of human
?Dr. J. B Roberts (Review), 6S.
Bright's disease?Dr. C. W. Purdy
(Review), 206.
Britain, The Races of?Dr. J. Beddoe
(Review), 64.
Brown, G. A.?A case of bronchial
croup, 115.
Calculi, Cases of vesical?C. F.
Pickering, 183.
Calculus on foreign body in bladder
?Dr. H. Benham and J. Greig
Smith, 38.
Carter, Dr. A. H.?Elements of
practical medicine (Revieiv), 141.
Chancre of the gum (Extract), 253.
Children, Surgical diseases of?E.
Owen (Review), 70.
Cocaine anaesthesia in major surgi-
cal operations (Extract) 121.
Cocaine, and some of its uses
(Extract), 196.
Cocaine, Injurious effects of?F. R.
Cross, 128
Colds, How to prevent (Extract),
200.
Cole, Dr. T.?Case of pernicious
anaemia, 231.
Comparative anatomy and physi-
ology?F. J. Bell (Review), 69.
Conduct, The springs of?Prof. C. L.
Morgan (Review), 212.
Contagious infection in surgery
(Extract), 122.
Corpulency, The treatment of (Ex-
tract), 46.
Cough, Reflex nasal (Extract), 194.
Crookshank, E. M.?Practical bac-
teriology (Review), 67.
Cross, F. R.?A case of poisoning by
duboisia, 127; injurious effects of
cocaine, 128; phlyctenular oph-
thalmia, 132; the surgery of the
upper eyelid, 186.
Croup, A case of bronchial?G. A.
Brown, 115.
Croup, Emetics in (Extract) 265.
Deformity resulting from extensive
loss of skin of arm, Operation to
correct (Extract), 124.
Dietary of the sick, Recent additions
to the?Dr. R. Shingleton Smith,
55-
Digest, The medical?Dr. R. Neale
(Review), 213.
Dislocation of ulna and radius?R.
J. H. Scott, 36.
Drainage in ascites, Permanent (Ex-
tract), 122.
296 INDEX.
Duboisia, A case of poisoning by?
F. R. Cross, 127.
Dysphagia of nervous origin (Ex-
tract), 266.
Ear, Diseases of the ? Dr. U.
Pritchard (Review), 284.
East, E.?The insane as single pa-
tients (Review), 281.
Elliott, Dr. C.?Case of intestinal
intussusception, 177.
Empyema, The treatment of?Dr.
W. H. C. Newnham, 225.
Epicystotomy (Extract), 119.
Epilepsy, Case of masked?Dr. B. B.
Fox, 172.
Epistaxis, The treatment of (Extract),
263.
Erysipelas, The antiseptic treatment
of (Extract), 251.
Eye, The refraction and accommo-
dation of the?Dr. E. Landolt
(Review), 209.
Eyelid, The surgery of the upper?
F. R. Cross, 186.
Facial neuralgia and dental irritation
?J. M. Ackland, 28.
Fairbanks, Dr. W.?The use of the
aspirator in retention of urine, 242.
Farquharson, Dr. R.?A guide to
therapeutics (Review), 142.
Fat, The formation of (Extract), 46.
Fehling's solution, Substitutes for
(Extract), 198.
Foetus in utero, On the?Dr. A.
Harvey (Review), 141.
Fox, Dr. B. B.?A case of masked
epilepsy, 172.
Fractures and dislocations?T. P.
Pick (Review), 70.
Freyer, Dr. P. J.? Litholapaxy
(Review), 277.
Frogskin, Grafts of (Extract), 256.
Galvanic battery, How to use a?
Dr. H. Tibbits (Review), 142.
Gastrotomy for removal of a fork
from the stomach (Extract), 254.
Germicides, So-called (Extract) 202.
Gerrard, A. W. ? Elements of
materia medica and pharmacy
(Review), 138.
Glaucoma (Extract), 204.
Gonorrhoea, 120, 121.
Gowers, Dr. W. R.?Diseases of the
brain (Review), Co.
Harley, Dr. G.?Inflammations of
the liver (Review), 139.
Harvey, Dr. A.?On the foetus in
utero (Review), 141.
Hemi-Rheumatism (Extract), 202.
Hernia, Radical cure of oblique
inguinal (Extract), 250.
Hiccough, Remedy for (Extract),
194- .
Hip-joint, Amputation at the (Ex-
tract), 120.
Hydatid disease, Treatment of
(Extract), 197.
Hydrocele, The radical cure of
(Extract), 124.
Hydrocephalus, A case of?Dr. W.
B. Roue, 102.
Hydrophobia (Extract), 260.
Hyoscine (Extract), 48.
Hysterical attack, Treatment of the
(Extract), 200.
Infant feeding (Extract), 200.
Insane as single patients, The?E.
East (Review), 281.
Insane in the United States and
Canada, The?Dr. D. H. Tuke
(Review), 68.
Intestinal canal, Passage of open
penknife along the (Extract), 254.
Intestinal injury, Case of emphy-
sematous abscess of thigh from?
Dr. G. H. Lilley, 181.
Intestinal intussusception, Case of?
Dr. C. Elliott, 177.
Intestinal malformation, Rare case
of (Extract), 125.
Intubation of the larynx in croup
(Extract), 252.
James, Dr. P.?A guide to the new
pharmacopoeia (Review), 142.
Jaundice, Catarrhal (Extract), 194.
Jessett, F. B.?Cancer of the ali-
mentary tract (Review), 275.
Kidney and lung, Operation on
(Extract), 256.
Kidney, The surgery of the?W. B.
Clarke (Review), 285.
Kidneys, Surgical diseases of?H.
Morris (Review), 69.
Landolt, Dr. E.?The refraction and
accommodation of the eye (Review),
209
Lansdown, F. P.?The progress of
practical surgery, 217.
INDEX. 2Q7
Laparotomy in strangulated hernia I Narium excoriationes?Dr. B. J.
[Extract), 249. I Baron, 52.
Laryngeal paralysis of central origin j Nasal bougies, Improved (Extract),
(Extract), 266. ! 265.
Laryngis, Chorea (Extract), 51, 266. 1 Nasal septum, Perforation of the
Laryngoscope in medicine, The? (Extract), 267.
Dr. B. J. Baron, 145. 1 Neale, Dr. R.?The medical digest
Larynx, Erysipelas of the (Extract), ! (Review), 213.
264; the treatment of tubercular j Nervous system, Diseases of?Dr. J.
ulceration of the (Extract), 262. Ross (Review), 66.
Lilley, Dr. G. H.?Case of emphy-
sematous abscess of thigh from | Ocular affections, The relation be-
intestinal injury, 181. | tween dental and (Extract), 203.
Litholapaxy?Dr. P. J. Freyer (Re- I (Esophagus, Resection of a cancer-
view), 277. j ous (Extract), 252.
Liver, Inflammations of the?Dr. G. ! Old age, The recuperative power of
Harley (Review), 139. j ?H. B. Runnalls, 168.
Lung and kidney, Case of operation ; Owen, E.? Surgical diseases of
on (Extract), 256. \ children (Review), 70.
Lungs, Diseases of the?Dr. D.Powell
(Review), 142. j Paraldehyde (Extract), 50.
Lupus, The treatment of (Extract), , Parasites of man, The?R. Leuckart
249. j (Review), 280.
Patella, Treatment of fracture of
MacCormac, Sir W.?Surgical oper- j (Extract), 126.
ations (Review), 67. j Pathology, Lectures on medical?
MacDonald, Dr. P. W. ? Heart-
lesions and mental symptoms,
162.
Maclaren, Dr. P. H.?Atlas of vene-
real diseases (Review), 68.
Macpherson, Dr. J.?Bath, Con
H. G. Sutton (Review), 273.
Pertussis (Extract), 199.
Pharmacopoeia, A guide to the new
?Dr. P. James (Review), 142.
Phlyctenular ophthalmia ? F. R.
Cross, 132
trexeville and the lime sulphated j Pick, T. P.?Fractures and disloca-
waters (Review), 137.
Mammary abscess, Abortive treat-
ment of (Extract), 255.
tions (Review), 70.
Pickering, C. F.?Cases of vesical
calculi, 183.
Materia medica and pharmacy, Ele- Pneumonia, Seasonal prevalence of
ments of?A. W. Gerrard (Review), , (Extract), 193.
1 }8. ' Penumonic abscess (Extract), 48.
Medicine, Elements of practical? ; Pneumotomy, Case of (Extract), 259.
Potassium bichromate, Action of
(Extract), 198.
Powell, Dr. D.?Diseases of the
lungs (Review), 142.
Practitioners, Unregistered (Review),
281.
Pregnancy, The vomiting of?Dr. J.
Meredith, 95.
Preparations for the sick, Notes on,
268.
Pritchard, Dr. U.?Diseases of the
Dr. A. H. Carter (Review), 141
Mental symptoms, Heart-lesions and
?Dr. P. W. MacDonald, 162.
Menthol in urticaria and pruritus
(Extract), 201.
Meredith, Dr. J.?The vomiting of
pregnancy, 95.
Microzymes in nutrition (Extract),
x33-
Morgan, Prof. C. L.?The springs of
Conduct (Review), 21
Morris H. ? Surgical diseases of ear (Review), 284.
kidneys (Review), 69. | Prostate, Diseases of the?Sir H.
Muscle by means of pedunculated Thompson (Review), 277.
muscle-flap, Elongation of (Ex- I
tract), 124. ! Quinsy as a rheumatism (Extract),
Myxoedema (Extract), 257. 1 265.
2gS INDEX.
Retinitis pigmentosa (Extract), 205. J Swayne, Dr. J. G.?A case of typh-
Roberts, Dr. J. B.?Operative sur- ) litis, 108.
gery of human brain (Review), 68
Rome in winter and the Tuscan hills
in summer?Dr. D.Young (Review),
Tendons, Reparation of (Extract),
123.
67. j Tetanus, Theory of origin of (Ex-
Ross, Dr. J.?Diseases of nervous j tract), 258.
system (Review), 66. ! Therapeutics, A guide to?Dr. R.
Roue, Dr. W. B.?A case of hydro- Farquharson (Review), 142.
cephalus, 102. j Thin, Dr. G.?Cancerous affections
Runnalls, H. B.?The recuperative of the skin (Review), 276.
power of old age, 168. j Thompson, Sir H.?Diseases of the
prostate (Review), 277.
Salol (Extract), 193. | Throat, Disagreeable sensations in
Schools, Infectious diseases in (Re- | the (Extract), 265.
view), 278. j Tibbits, Dr. H.?How to use a
Sciatica (Extract), 201. ! galvanic battery (Review), 142.
Scott, R. J. H.?Dislocation of ulna | Tonic contraction of lower extremi-
and radius, 36. I ties?W. H. Webb, 42
Scraps, 71, 143, 215, 287.
Sex in disease, The influence of?
W. R. Williams (Review), 68.
Sharkey, Dr. S. J.?Spasm in chronic
nerve disease (Revieiv), 283.
Skin, Cancerous affections of the?
Dr. G. Thin (Review), 276.
Smith, Dr. R. Shingleton?A case
of aneurism of aorta, 233; recent |
additions to the dietary of the : Urethan (Extract), 50.
sick, 55. I Uterine appendages, Removal of
Smith, J. Greig?Calculus on foreign ; the?J. Greig Smith, 1, 73.
body in bladder, 38 ; removal of 1
the uterine appendages, 1, 73.
Sodium sulphate as an aperient
Tracheotomy, Ulceration of the
trachea and innominate artery
after (Extract), iiq.
Tuke, Dr. D. H.?The insane in the
United States and Canada (Re-
view), 68.
Typhlitis, A case of ? Dr. J. G.
Swayne, 108.
o I%" -i c r. ! Vertebrae, Operations on the (Ex-
South, J.F.?Memorials of the craft tract) ->48
of surgery (Review), 274. : Voc'al ^r4ds from hili Uniti
Spasm in chronic nerve disease? j f th (Extnict)
Dr. S. J- Sharkey (Review), 283. ; Vocal cordl Syphiloma of both (^-
Stomach diseases, New methods ol j
treating (Extract), 47. J '
Strophantin (Extract), 51. . .
Surgery, A manual of?Edited by | Webb, W. H.?-Ionic contraction of
F. Treves (Review), 140. | lower extremities, 42.
Surgery, Memorials of the craft of? J Williams, vv. R. I he influence of
J. F. South (Review), 274.
Surgery, The progress of practical?
F. P. Lansdown, 217.
Surgical operations?Sir W. Mac
Cormac (Review), 67.
Sutton, H. G.?Lectures on medical
pathology (Review), 273.
Venereal diseases, Atlas of?Dr. P
H. Maclaren (Review), 68.
sex in disease (Review), 68.
Wise, A. T.?Alpine winter (Review),
142.
Young, Dr. D.?Rome in winter and
theTuscan hills in summer (Review),
67.
J. W. ARKOWSMITH, PRINTER, QUAY STREET, BRISTOL.

				

